# Clonal Waves of *Neisseria* Colonisation and Disease in the African Meningitis Belt: Eight- Year Longitudinal Study in Northern Ghana

**DOI:** 10.1371/journal.pmed.0040101

**Published:** 2007-03-27

**Authors:** Julia Leimkugel, Abraham Hodgson, Abudulai Adams Forgor, Valentin Pflüger, Jean-Pierre Dangy, Tom Smith, Mark Achtman, Sébastien Gagneux, Gerd Pluschke

**Affiliations:** 1 Swiss Tropical Institute, Basel, Switzerland; 2 Navrongo Health Research Centre, Ministry of Health, Navrongo, Ghana; 3 Max Planck Institute for Infection Biology, Berlin, Germany; Centers for Disease Control, United States

## Abstract

**Background:**

The Kassena-Nankana District of northern Ghana lies in the African “meningitis belt” where epidemics of meningococcal meningitis have been reoccurring every eight to 12 years for the last 100 years. The dynamics of meningococcal colonisation and disease are incompletely understood, and hence we embarked on a long-term study to determine how levels of colonisation with different bacterial serogroups change over time, and how the patterns of disease relate to such changes.

**Methods and Findings:**

Between February 1998 and November 2005, pharyngeal carriage of Neisseria meningitidis in the Kassena-Nankana District was studied by twice-yearly colonisation surveys. Meningococcal disease was monitored throughout the eight-year study period, and patient isolates were compared to the colonisation isolates. The overall meningococcal colonisation rate of the study population was 6.0%. All culture-confirmed patient isolates and the majority of carriage isolates were associated with three sequential waves of colonisation with encapsulated (A ST5, X ST751, and A ST7) meningococci. Compared to industrialised countries, the colonising meningococcal population was less constant in genotype composition over time and was genetically less diverse during the peaks of the colonisation waves, and a smaller proportion of the isolates was nonserogroupable. We observed a broad age range in the healthy carriers, resembling that of meningitis patients during large disease epidemics.

**Conclusions:**

The observed lack of a temporally stable and genetically diverse resident pharyngeal flora of meningococci might contribute to the susceptibility to meningococcal disease epidemics of residents in the African meningitis belt. Because capsular conjugate vaccines are known to impact meningococcal carriage, effects on herd immunity and potential serogroup replacement should be monitored following the introduction of such vaccines.

## Abstract 2

An analysis of pharyngeal carriage of meningococci in one district of Ghana examined the features of the isolates that might contribute to the susceptibility to meningococcal epidemics in the African meningitis belt.

## Editors' Summary

**Background.:**

Bacterial meningitis is a rare but often fatal infection of the meninges—the thin membrane around the brain and the spinal cord. It can be caused by several types of bacteria, but meningococcal meningitis, which is caused by *Neisseria meningitidis,* is the most common form of bacterial meningitis in children and the second most common form in adults. About 10% of healthy people have N. meningitidis growing in their nose and throat; the bacteria are spread by exposure to infected respiratory secretions. In these “carriers,” the immune system keeps the bug in check but sometimes this surveillance fails, N. meningitidis enters the bloodstream and travels to the brain, where it infects the meninges and causes inflammation. The symptoms of meningococcal meningitis are sudden fever, headache, and a stiff neck and, even if strong antibiotics are given quickly, 10%–15% of patients die.

**Why Was This Study Done?:**

Outbreaks of meningococcal meningitis occur all over the world, but the highest burden of disease is in the African meningitis belt, which stretches across sub-Saharan Africa from Senegal to Ethiopia. Here, localized epidemics of meningococcal meningitis occur every eight to 12 years during the dry season. Control of these epidemics relies on their early detection followed by mass immunization. This approach can be hard to implement in countries with limited resources, but the introduction of other control measures (for example, routine childhood immunization) requires an understanding of how the spread of different strains of N. meningitides through the community causes periodic epidemics. In this study, the researchers have studied the long-term dynamics of colonization by N. meningitidis and the occurrence of meningococcal meningitis in one region of the African meningitis belt.

**What Did the Researchers Do and Find?:**

The researchers took throat swabs twice a year from people living in rural northern Ghana for eight years. They tested each swab for N. meningitidis and determined the serogroup of the bacteria they found. Bacterial serogroups differ only in terms of the antigens (molecules recognized by the immune system) that they express; N. meningitidis is classified into 13 serogroups based on the sugars that coat its surface. The researchers also used DNA sequencing to group the bacterial isolates into genoclouds—genetically closely related groups of meningococci represented by a sequence type (ST) number. Finally, they monitored meningococcal disease throughout the study and determined the serogroup and genocloud of patient isolates. Their results show colonization of 6% of the study population by N. meningitidis and reveal three consecutive waves of colonization and disease characterized by the presence of a serogroup A ST5 genocloud, a serogroup X ST751 genocloud, and finally a serogroup A ST7 genocloud. Colonizing bacteria isolated in this study in Ghana, the researchers report, changed their genotype more frequently but were less genetically diverse than those isolated in industrialized countries. In addition, the commonest serogroups of N. meningitidis in carriers in Ghana were disease-causing serogroups, whereas in industrialized countries these serogroups are rarely seen in carriers. However, non-groupable bacteria (bacteria that lack surface sugars), although common in industrialized countries, were rare in Ghana.

**What Do These Findings Mean?:**

These findings begin to explain why epidemics of meningococcal meningitis are common in the African meningitis belt. Because there isn't a stable, genetically diverse population of N. meningitidis in carriers, the immune systems of people living here may not be optimally prepared to deal with new bacterial clones that arrive in the region, and this lack of immunity could result in frequent epidemics. However, because the researchers took relatively few samples every six months from one small area of the meningitis belt, the genetic diversity of N. meningitidis over the whole region might be considerably greater than that colonizing the study population. Nevertheless, the description of successive waves of meningococci colonization in Ghana has important implications for the proposed introduction of childhood vaccination against meninogococcal disease in the African meningitis belt. If this vaccination program goes ahead, warn the researchers, it will be essential to monitor which strains of N. meningitidis are colonizing the population and to have emergency plans ready to deal with any emerging disease-causing serogroups that are not covered by the vaccine.

**Additional Information.:**

Please access these Web sites via the online version of this summary at http://dx.doi.org/10.1371/journal.pmed.0040101.

- The Web sites of the institutions at which this research was performed, the Swiss Tropical Institute and the Navrongo Health Research Centre, provide more information about the programs
- The World Health Organization provides information on meningococcal disease, including the African meningitis belt (in English, Spanish, Chinese, Russian, and Arabic)
- Information on meningitis and vaccines and their potential use in Africa is available from the Meningitis Vaccine Project (in English and French)
- Medline Plus has encyclopedia pages on meningococcal meningitis
- The US Centers for Disease Control and Prevention provides information on meningococcal disease (in English and Spanish)

## Introduction

The highest burden of meningococcal meningitis occurs in the “meningitis belt” of sub-Saharan Africa, a region stretching from Senegal to Ethiopia with an estimated population of 300 million [1,2]. Within individual areas of the meningitis belt, major disease epidemics occur in irregular cycles every eight to 12 years, with attack rates ranging from 100 to 1,000 per 100,000 population [3]. Epidemics start in the early dry season, stop abruptly at the onset of the rains, but may break out again in the following dry season. Low humidity and high temperatures may favour the occurrence of meningococcal disease by damaging mucosal surfaces and the immune defence. In any one country, epidemics occur over a period of only two to three years [1]. The periodicity of these epidemics is not well understood, nor is it possible to predict them accurately. The current approach for control of meningococcal disease epidemics is based on early detection of the disease by the epidemic threshold of ten to 15 cases per 100,000 inhabitants per week [4] followed by mass immunisations with polysaccharide vaccines [3]. However, in settings with limited resources, effective surveillance and timely interventions are difficult to implement, and therefore vaccination campaigns are often delayed [1].

N. meningitidis can be classified into 13 serogroups on the basis of the chemical composition of its polysaccharide capsule [5]. Serogroup A accounts for most epidemics in the African meningitis belt, but C and W135 epidemics have also been reported [1,6]. Meningococci that cause epidemics are genetically closely related; specific genotypes plus their epidemiologically associated genetic descendants constitute “genoclouds” [7]. The two most recent meningococcal disease pandemics originated in Asia and were caused by serogroup A meningococci belonging to two related genoclouds [7]. These two genoclouds have been assigned the sequence type (ST) 5 and ST7, respectively, based on multilocus sequence typing [7,8]. At the time we started our study serogroup W135 meningococci was considered a rare cause of invasive disease. However, two recent W135 meningitis outbreaks in Mecca were followed by major epidemics in Burkina Faso [6,9].

N. meningitidis is a commensal of the human nasopharyngeal mucosa. It is transmitted by aerosol droplets or through contact with respiratory secretions. Because meningococcal transmission is independent of disease, characterisation of the carrier state is crucial for understanding the epidemiology of meningococcal disease. Multiple colonisation studies have been performed in industrialised countries, but little is known about the meningococcal colonisation dynamics in Africa. Here, we report the findings of the first (to our knowledge) long-term colonisation study carried out in the African meningitis belt.

## Methods

### Study Area

The study was conducted in the Kassena-Nankana District (KND) of the Upper-East Region of Ghana. It lies within the Guinea savannah woodland and has two major seasons: a short wet season from June to October and a long dry season for the rest of the year. The district population is about 140,000, mainly rural, except for the 20,000 inhabitants of Navrongo town. In the KND, people live in compounds with an average of ten inhabitants [10]. Between 1997 and 2002, yearly vaccination campaigns with meningococcal serogroup A/C polysaccharide vaccine targeted the whole district population. Between 2003 and 2005, smaller campaigns were carried out. In 2003, 80% of the study participants were reported to have been vaccinated within the previous three years.

### Ethical Approval and Consent

Ethical clearance for this study was obtained from the relevant institutional review boards and the Ghana Ministry of Health. Informed consent was obtained from all study participants.

### Colonisation Isolates

We randomly selected 37 residential compounds from a complete listing of the district population using the Navrongo Demographic Surveillance System (NDSS) [11]. The sample size was chosen to include a total population of about 300 individuals per survey, which means that a carriage rate of 5% at any one survey (corresponding to relatively frequent infection) can be distinguished at a significance level of 5% from those of infrequent infections carried by only 2.5% of the population.

Throat swabs were taken twice annually from all inhabitants of the 37 compounds present at the time of the visit who agreed to participate. A total of 16 surveys have been performed since March 1998; in each of them between 292 and 350 study participants have been swabbed (Table 1). The number of participants swabbed in single compounds ranged from zero to 30 (mean 8.57, median 6); fluctuations in these numbers were due to travelling activities. Three individuals refused to participate in the study; as a result one of the compounds was discontinued after the first visit. Another compound was replaced in April 2002 after being deserted by its inhabitants. The age distribution of the study participants was comparable with the overall age distribution in the KND (Table 2).

**Table 1:**
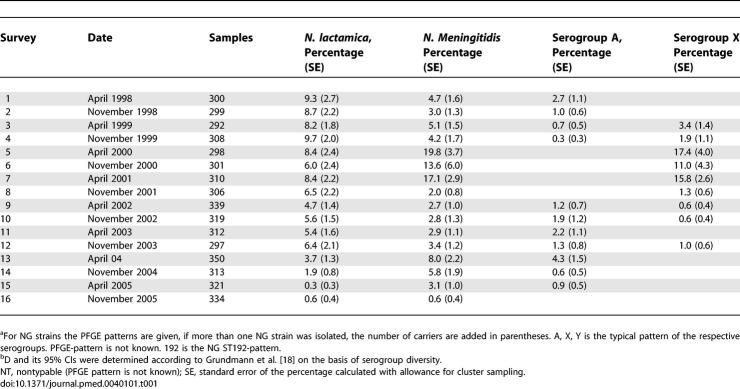
Carriage Rates in Percentage during 16 Carriage Surveys in the KND

**Table 1:**
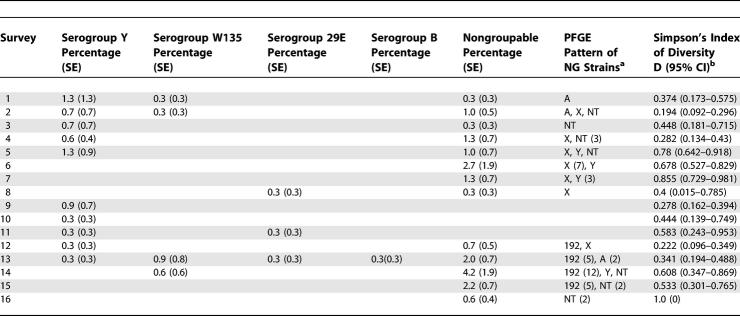
Continued

**Table 2:**
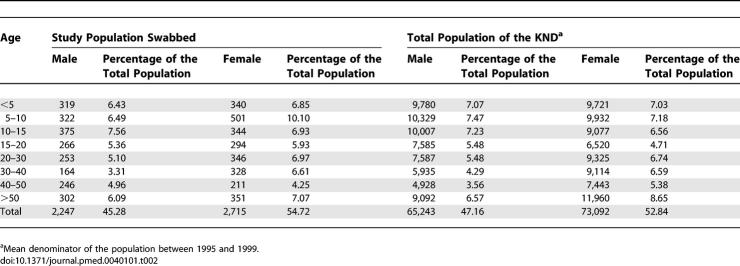
Age Distribution of the Overall Study Population during Eight-Year Colonisation Surveys in Comparison with the Overall Population in the KND According to Navrongo Demographic Surveillance System Surveys 1995–1999

At the compound visit, each throat swab was taken and directly inoculated on Thayer-Martin agar plates [12]. From each positive plate, two colonies with neisserial morphology were subcultured. N. meningitidis and N. lactamica colonies were identified by standard bacteriological methods as previously described [12].

### Disease Isolates

Patients with suspected meningitis presenting at the War Memorial Hospital, Navrongo, or one of the four health centres of the KND were recruited throughout the study period. Suspected meningitis was defined by sudden onset of fever and stiff neck in the patient, or fever and stiff neck and altered mental status, in accordance with World Health Organization guidelines [3]. A lumbar puncture was performed before treatment on all patients with suspected meningitis, and the cerebrospinal fluid specimen was analysed in the laboratory of the War Memorial Hospital as described previously [12]. In 1998–1999, only samples collected during the dry season were analysed. Thereafter, samples collected from the few patients with suspected meningitis who presented during the wet season were also included.

### Characterisation of Bacterial Isolates

Meningococci were serogrouped with serogroup-specific antisera (Difco, http://www.bd.com) according to the manufacturer's instruction. In a subset of isolates, serological typing was confirmed by PCR [13,14]. All isolates were analysed by pulsed-field gel electrophoresis (PFGE) after digestion of genomic DNA with NheI [15]. Multilocus sequence typing was performed as described [8].

### Statistical Methods

As a measure of the invasiveness of the different genoclouds, we computed the ratio of cases to the approximate number of carriers in the district (Table 3). To estimate the latter we assumed that each survey was representative of the six-month period in which it was carried out, and that average duration of carriage is six months, corresponding approximately to the half-life of N. meningitidis carriage determined in a study in university students in the United Kingdom [16]. The diversity index (D) (Table 1) of the meningococcal carriage population was determined with respect to the serogroup distribution for each of the 16 surveys and for the overall study period using the Simpson index [17,18].

**Table 3:**
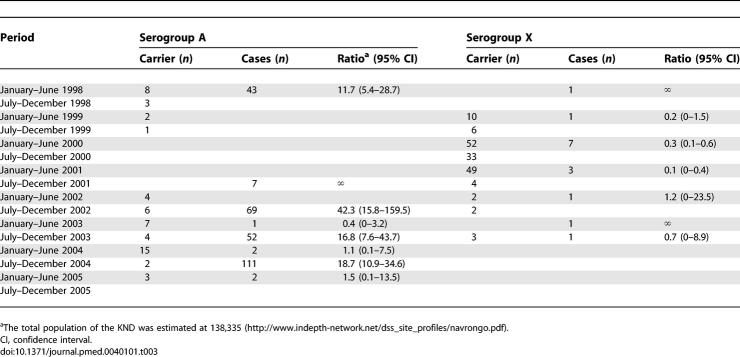
Ratio of Meningitis Cases Versus 1,000 Carriers for Serogroup A and X at Different Time Points

The unadjusted proportion of samples carrying each bacterium provides a consistent and unbiased estimate of carriage prevalence; however, because of the random sampling of the population, the clustering in the sampling procedure introduced extrabinomial variation, which was allowed for in the estimates of standard errors (Table 1) by calculating robust (sandwich) estimates [19] using Stata version 8.0 (Stata Corporation, http://www.stata.com).

Risk ratios and confidence intervals (CIs) were calculated using EpiInfo version 3.3.2 (http://www.cdc.gov/EpiInfo), but these analyses treated the different samples as statistically independent, and consequently some of the tests carried out are anticonservative because of the effects of repeated sampling of the same individuals. We draw attention to the implications of this repeat sampling where applicable.

## Results

### Clonal Waves of Meningococcal Colonisation and Disease

**Figure 1:**
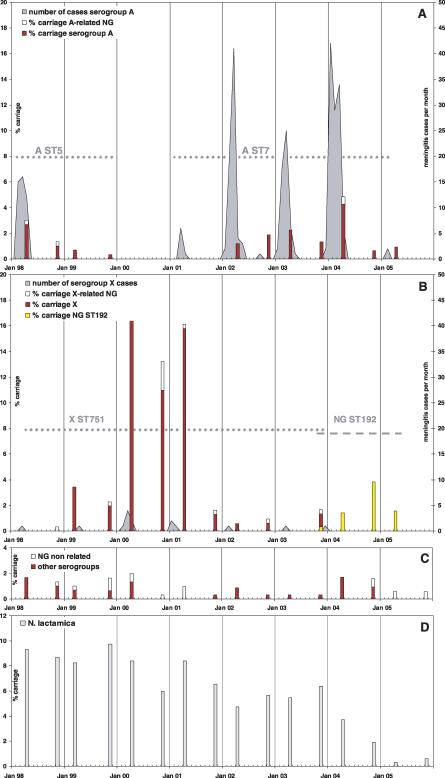
Waves of Colonisation and Disease in the KND from April 1998 until November 2005 Carriage rates recorded during 16 colonisation surveys (April and November each year) and monthly numbers of confirmed meningitis cases of *N. meningitidis.* (A) Genoclouds of serogroup A ST5 and ST7 meningococci are shown. (B) Genoclouds of serogoup X ST851 and NG ST192 meningococci are shown. (C) Carriage rates of other serogroups and meningococci unrelated to the A, X, or NG ST192 genoclouds are shown. (D) Carriage rates of N. lactamica are shown.

We monitored the dynamics of pharyngeal carriage of N. meningitidis and bacterial meningitis in the KND of northern Ghana from February 1998 to November 2005; three major waves of clonal colonisation and disease with encapsulated meningococci were observed. A meningitis epidemic occurred in the dry season of 1996–1997 with 1,396 suspected meningitis cases in the KND, but because of lack of infrastructure no laboratory analysis was performed [20]. This epidemic was followed by a smaller outbreak with 50 laboratory-confirmed serogroup A meningitis cases in the following dry season. A total of 36 isolates were culture confirmed and identified as subgroup III, ST5 bacteria [12], which spread throughout the meningitis belt after an epidemic in Mecca in 1987 [21]. Carriage of serogroup A ST5 meningococci decreased steadily from 2.7% (8/301) in April 1998 to 0.3% (1/308) in November 1999 (Figure 1A). Thereafter, none of the clinical or colonisation isolates from the KND belonged to the serogroup A ST5 genocloud. In 2000, no serogroup A meningococci were isolated from either patients or carriers. However, in 2001, a new wave of serogroup A meningococcal colonisation and disease started. All serogroup A carrier and disease strains isolated since then belonged to a new genocloud of serogroup A meningococci associated with ST7 that was observed for the first time in Africa in 1995 [7]. Although colonisation was still low in April 2001 (i.e., <0.3%), seven serogroup A ST7 meningitis cases were identified between February and March 2001. In the following three years, serogroup A ST7 colonisation rates of 1.2%–4.3% were observed. Despite yearly serogroup A/C polysaccharide mass immunisations, this low level of colonisation was associated with repeated serogroup A ST7 meningitis outbreaks in the KND (Figure 1A). Laboratory-confirmed cases numbered 70 between January and May 2002, 56 were identified between January and May 2003, and 113 were identified between December 2003 and April 2004. Thereafter, the serogroup A ST7 colonisation rate dropped below 1%, and only two serogroup A ST7 meningitis cases were recorded in February 2005.

Between the two waves of serogroup A colonisation and disease, we documented a wave of colonisation with a serogroup X ST751 genocloud (Figure 1B) [22,23]. The extensive spread of this low-virulence serogroup was associated with a total of 15 meningitis cases between 1998 and 2003. Serogroup X carriage and disease peaked in the dry seasons of 1999/2000 and 2000/2001, with colonisation rates of 17.3% and 15.1%, respectively. While in the peak of the serogroup X wave the ratio of serogroup X cases to carriers was found to be between 0.1 and 0.3:1,000 (Table 3), and the observed ratio of serogroup A cases versus carriers during the A ST7 outbreak was between 16.8 and 42.3:1,000 in the respective dry seasons.

Since November 2003, 23 non(sero)groupable (NG) ST192 carriage isolates with closely related PFGE patterns were collected (Figure 1B). Their colonisation rate peaked in November 2004 at 3.8% (12/313). NG ST192 strains isolates have been previously reported from The Gambia and Niger (for listing, see http://pubmlst.org/perl/mlstdbnet/mlstdbnet.pl?page=st-query&file=pub-nm_isolates.xml).

### Investigation of Patients with Meningitis

Throughout the study period, 1,145 suspected meningitis patients were recruited and cerebrospinal fluid samples were analysed in the lab. Overall, 311 meningococcal meningitis cases were confirmed by culture and/or latex agglutination. Furthermore, a pneumococcal meningitis outbreak with serotype 1 dominating occurred between 2000 and 2003 [24], while overall only 17 Haemophilus influenzae meningitis cases were reported. We obtained meningococcal isolates in 197/311 (63%) of confirmed cases.

Latex agglutination confirmed the serogroup A capsule for all 114 cerebrospinal fluid samples that were negative in culture. All recovered disease isolates belonged to the three dominating genoclouds of encapsulated meningococci (36 serogroup A ST5, 148 serogroup A ST7, and 15 serogroup X strains). With respect to colonisation, 289 (5.8%) of the pharyngeal samples contained N. lactamica and 302 (6.0%) *N. meningitidis.* Point prevalence of the different serogroups at the 16 individual surveys is given in Table 1. All serogroup A (*n* = 55) and serogroup X (*n* = 161) carriage isolates belonged to the three genoclouds causing the major sequential colonisation waves. In addition, 16 NG isolates shared ST and PFGE patterns with the isolates of serogroup A ST5 (two isolates), serogroup A ST7 (two isolates), or serogroup X (12 isolates), respectively (Figure 1A). These colonisation isolates thus represented unencapsulated variants of the respective genoclouds. There was no evidence for an accumulation of the nonencapsulated variants toward the end of the colonisation waves (Figure 1). In some cases, encapsulated and NG variants of the same genocloud were found simultaneously in the same compound. Bacterial carriage showed only moderate clustering by compound. Of the 39 compounds sampled at least once, 31 (79%) provided at least one N. lactamica and 36 (92%) at least one N. meningitidis positive sample. Of the 583 individual compound visits, 158 (27%) yielded N. lactamica and 155 (27%) N. meningitidis.

To compare these numbers with those expected if the bacteria were randomly distributed between compounds, we selected 289 of the samples in the database at random and found that 208 different visits including 38 compounds were represented, while a different, independent set of 302 samples chosen at random corresponded to 213 visits to 37 of the compounds.

The diversity index D (Table 1) varied strongly between the single-sampling time points. Highly homogenous carriage populations were found in the course of colonisation waves with peak values of D = 0.855 in April 2001 for the X wave, and D = 0.583 in April 2003 for the A ST7 wave. In contrast, carriage isolates were much more diverse in the transition phase between two clonal waves with the lowest values of D = 0.194 in November 1998 during the transition from A ST5 to X, and D = 0.278 in April 2002 during the transition from X to A ST7. Overall, the serogroup diversity in the KND (D = 0.349, 95% CI 0.306–0.393) was slightly lower than in a carriage study in Europe (D = 0.282, 95% CI 0.251–0.313) [25].

### Low Background of Meningococci Unrelated to the Clonal Waves

Only from 16.6% (50/302) of the meningococcal carriers were colonisation isolates unrelated to the dominating serogroup A, X, and NG ST192 genoclouds (Figure 1C). Although neighbouring Burkina Faso was hit by repeated W135 ST11 epidemics in the dry seasons of 2002–2004, in the KND carriers of the epidemic strain were only found in April 2004 (3/350; 0.9%) and November 2004 (2/313; 0.6%), and not a single W135 meningitis case was recorded between 1998 and 2005 [26]. Single carriers of W135 ST11 meningococci were also identified in April (1/300) and in November 1998 (1/299) [23], two years prior to a first documented W135 meningitis outbreak in Mecca [9]. While serogroup Y meningococci (21 isolates) and serogroup Y ST168 related NG strains (seven isolates) were isolated in ten out of the 16 individual surveys, carriage of serogroup B and serogroup 29E meningococci was rare (Table 1). Carriage of serogroup Y meningococci was strongly associated with one particular compound, where during eight of the 16 surveys, 67% (14/21) of the serogroup Y strains were isolated. Altogether, only eight NG isolates had PFGE patterns and STs unrelated to the dominating serogroup A, X, Y, and NG ST192 genoclouds (Table 1). While the N. lactamica carriage rate remained relatively constant (4.7%–9.3%) for six years, it declined after April 2004 to 0.3% in April 2005 (Figure 1D). We observed no significant association between the A/C meningococcal polysaccharide vaccine immunisation status and meningococcal carriage of all serogroups (relative risk [RR] = 1.11; *p* = 0.81; 95% CI 0.65–1.95), of serogroup A (RR = 0.9; *p* = 0.92; 95% CI 0.42–1.88), or of N. lactamica (in the >2-year-old RR = 0.7; *p* = 0.3; 95% CI 0.37–1.27). These CIs do not allow for the effect of clustering in the sampling. An analysis that allowed for clustering would give even higher *p*-values, confirming the absence of effect.

### Age Distribution of Carriers and Patients

**Figure 2:**
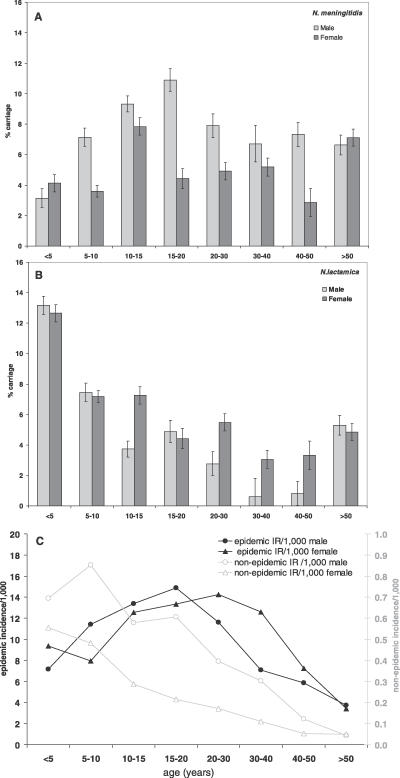
Age and Sex Patterns of Colonisation and Disease (A) Carriage of meningococci (all serogroups and NG; cumulation of all surveys) in the different age groups of the male (light grey bars) and female (dark grey bars) population are shown. 95% CIs are indicated. These CIs do not allow for repeated sampling. (B) Carriage of N. lactamica in the different age groups (mean over all surveys) of the male (light grey bars) and the female population (dark grey bars) are shown. 95% CIs are indicated. These CIs do not allow for repeated sampling. (C) Age spectrum of IR of meningococcal meningitis in the male (circles) and female (triangles) population of the KND in the epidemic of 1996–1997 (dark grey) versus the interepidemic period 2001–2005 (light grey). Denominator is the district population 1995–1999. On the primary y-axis the epidemic IRs and on the secondary y-axis the interepidemic IRs are indicated.

Colonisation with meningococci in the KND exhibited a broad age range (Figure 2A). It peaked in teenagers and young adults (median age 17.9 y; range 5 mo–84 y). In contrast, the carriage rate of N. lactamica was highest in the <5-y age group (Figure 2B). During the 1996/1997 epidemic the age pattern of clinically diagnosed meningitis patients (median age 17.8 y; range 3 mo–80 y) resembled that of meningococcal carriers (Figure 2C), the incidence rates (IRs) of males (*n* = 628; IR = 9.5 per 1,000 population) and females (*n* = 713; 9.8 per 1,000) were comparable (RR = 0.97; *p* = 0.59). In contrast, during the postepidemic serogroup A meningococcal disease outbreaks between 1998 and 2005, the incidence of meningitis was highest in children <10 y of age and decreased steadily with age (Figure 2C). The median age of A ST5 cases in 1998 and of A ST7 cases in 2001–2005 was comparable (8.0 y; range 4 mo–64 y versus 10.0 y; range 2 mo–75 y, respectively). However, between 2001 and 2005 the IR of males (*n* = 159; IR = 0.49 per 1,000) was significantly higher (RR = 2.0; *p* < 0.001) than of females (*n* = 89; IR = 0.24 per 1,000). The case fatality rate of A meningococcal meningitis was much higher during the A ST5 postepidemic outbreak in 1998 (20%; 10/50) than during the epidemic in 1996–1997 (4.7%; 65/1396; *p* < 0.001) or during the A ST7 outbreaks in 2001–2005 (4.8%; 11/238; *p* < 0.001).

## Discussion

Here, we present the results of the first longitudinal study (to our knowledge) on meningococcal colonisation and disease in the meningitis belt of sub-Saharan Africa. The study revealed features that are in many aspects remarkably different from findings of colonisation studies conducted in Europe and North America [5,27–30]. The population of meningococci carried in the KND (i) was less constant in genotype composition; (ii) was less genetically diverse during the peaks of colonisation waves; (iii) included fewer NG strains; and (iv) was characterised by dominant virulent encapsulated strains. Indeed, the A ST5, A ST7, and X ST751 meningococci responsible for all 197 culture-reconfirmed meningitis cases represented 71% (216/302) of the colonisation isolates.

These results allow us to describe the major developments in meningococcal carriage and disease in the KND over an eight-year study period that included both meningococcal outbreaks and the interepidemic periods. We recorded very good compliance of the study participants. However, financial and organisational constraints did not allow sampling intervals shorter than six months, and this sampling interval may have been too long for a robust analysis of acquisition rates and duration of carriage. The relatively small number of 300 study members also led to a rather low sensitivity and thus to the late detection of the serogroup A ST7 colonisation in the district. Meningococcal colonisation with minor clones might also have been undetected.

In industrialised countries, approximately 10% of individuals from the general population are carrying meningococci at any one time [31]. In children younger than four years, carriage rates are less than 3%. They increase to 20%–40% in teenagers and young adults [27,31–33] and decrease again to less than 10% in older age groups. In contrast, invasive meningococcal disease is most common in young children and in teenagers. Current annual incidence of endemic meningococcal disease in most industrialised countries ranges from less than one to five cases per 100,000 population. In industrialised settings, meningococcal strains collected from patients and carriers differ genetically and serologically [27]. Typically, the populations of meningococci carried are highly diverse, with a low representation of the invasive serogroups A, B, C, Y, and W135 [5,28–30]. The genetic composition of the strains carried is relatively constant over time, and up to 50% are serologically nongroupable [5,31]. Encapsulation is thought to reduce adherence to pharyngeal epithelial cells, and loss of expression of capsular polysaccharide may be an adaptation to long-term carriage [34]. Colonisation with NG strains may be beneficial to the host by eliciting cross-reactive immune responses to noncapsular meningococcal surface antigens [34].

The observed lack of a stable and genetically diverse resident pharyngeal flora of meningococci in the KND may explain why incoming new clones can spread so successfully in populations of the African meningitis belt. The instability in the resident flora evidently leads to clonal waves of colonisation typically lasting for about four years and, in the case of hypervirulent lineages, disease outbreaks or epidemics. We found that the case-to-carrier ratio was generally much higher for serogroup A than for serogroup X meningococci, reflecting the marked difference in virulence between these two serogroups. Only in the dry season of 2001 at the beginning of the A ST7 colonisation and disease wave did we find patient isolates that were unrepresented during the corresponding colonisation survey. The highest A ST7 colonisation rate (4.3% in April 2004) was associated with the largest meningococcal meningitis outbreak observed during the entire study period. These data give no strong indication for a change in the case-to-carrier ratio in the course of the serogroup A ST7 outbreak.

New contact of the population with genoclouds that have epidemic potential does not always lead to high colonisation rates. For example, we recovered isolates resembling those responsible for the 2002–2004 epidemics in Burkina Faso from a few carriers in KND in 2004, but we did not observe any waves of W135 colonisation. Fluctuations of pharyngeal microflora of the population, however, are not confined to the meningococci. For example, the N. lactamica colonisation rate also changed in the course of the study. In addition, an outbreak of pneumococcal meningitis occurred during the study period with features (seasonality, clonality, and a broad age spectrum) characteristic of meningococcal epidemics [24]. Increasing herd immunity may be responsible for the disappearance of dominating genoclouds. However, changes in herd immunity do not fully explain the complete disappearance of the A ST5 genocloud two years after the 1996/1997 epidemic, nor the emergence of the closely related A ST7 genocloud after only a short time interval.

The age distribution of healthy carriers in the KND with peak carriage rates in teenagers and young adults was similar to that found in many European colonisation studies [5,31,33]. The incidence of meningitis during the disease outbreaks in the years 1998–2005 was highest in children less than ten years old, comparable to endemic disease in industrialised countries. It is thought that immune responses elicited by colonisation with meningococci and other antigenically cross-reactive microorganisms are responsible for the decreased disease susceptibility in the older age groups. This difference in age pattern between colonisation and disease may imply that natural serum antibody-mediated immunity against invasive disease develops much more efficiently than secretory IgA-mediated protection against colonisation.

However, during the epidemic in 1996–1997, the age distribution of meningitis patients resembled that of meningococcal colonisation, consistent with reports of most large meningococcal epidemics [1,2,35,36]. During the epidemic the disease susceptibility of the whole population was increased, and an overall IR of 9.7 per 1,000 was recorded. The fact that the epidemic incidence of meningitis dramatically exceeded endemic attack rates also in children less than ten years old, argues against the “two hit” hypothesis, that susceptibility to disease is associated with blocking serum IgA elicited by colonisation of the gut with cross-reactive microorganisms [37].

The factors that initiate epidemics in the meningitis belt are incompletely understood. Contact of a population with a hyperinvasive new genocloud that is antigenically distinct enough to escape natural immunity may lead to an epidemic. Loss of natural immunity in exposed individuals over time and new birth cohorts may make a population increasingly susceptible. However, epidemics are not always associated with the appearance of a new clone [1]. This suggests a role of environmental triggers, such as.periods of hot, dry, and dusty weather, copathogens, or social factors. Despite intense annual A/C polysaccharide vaccination campaigns carried out in the KND since 1998, outbreaks with IRs of up to 80 per 100,000 occurred between 2002 and 2004. Even though, this incidence was less than one-tenth of the disease burden during the 1996–1997 epidemic in the KND, it was still several times higher than in industrialised countries [38]. It is not clear whether herd immunity elicited by the serogroup A ST5 epidemic, lack of environmental triggers, or the vaccination campaigns have prevented a large A ST7 epidemic.

Meningococcal vaccines protect individuals from disease by eliciting bactericidal serum antibodies [39]. Recent studies following the introduction of conjugate C vaccines in the United Kingdom have demonstrated that capsule conjugate vaccines also affect carriage and transmission by inducing mucosal immune responses [40,41]. Herd immunity may play a key role in the control of meningococcal infection using meningococcal conjugate vaccines [42]. An affordable serogroup A conjugate vaccine may soon become available and introduced in the African meningitis belt (http://www.meningvax.org). Serogroup replacement and the emergence of escape variants [43] are potential consequences of limited-spectrum vaccines, but these factors cannot be well understood without carriage studies. Our data suggest that successive waves of meningococci replace each other in the meningitis belt, some leaving little trace in disease surveillance statistics because of their low invasiveness. Once conjugate vaccines are introduced it will be critical to ensure that there is no replacement of vaccine serogroups with invasive alternatives. Carriage studies will play an important role in monitoring bacterial dynamics in order to anticipate any such problems and to prepare responses, such as mass vaccination with a supplementary carbohydrate vaccine.

## Abbreviations

CI: confidence interval
D: diversity index
IR: incidence rate
KND: Kassena-Nankana District
NG: non(sero)groupable
PFGE: pulsed field gel electrophoresis
RR: relative risk
ST: sequence type
