# Correction: Clonal Waves of Neisseria Colonisation and Disease in the African Meningitis Belt: Eight-Year Longitudinal Study in Northern Ghana

**DOI:** 10.1371/journal.pmed.0040196

**Published:** 2007-05-29

**Authors:** Julia Leimkugel, Abraham Hodgson, Abudulai Adams Forgor, Valentin Pflüger, Jean-Pierre Dangy, Tom Smith, Mark Achtman, Sébastien Gagneux, Gerd Pluschke

In *PLoS Medicine*, volume 4, issue 3, doi: 10.1371/journal.pmed.0040101:

The text of the Acknowledgments section was incomplete. It should have read: “We acknowledge the use of the Neisseria Multi Locus Sequence Typing Web site (http://pubmlst.org/neisseria) developed by Keith Jolley and Man-Suen Chan and sited at the University of Oxford. The development of this site has been funded by the Wellcome Trust and European Union. We are grateful for support and contributions of E. Arnold, F. Binka, S. Droz, I. Ehrhard, B. Genton, and M. Tanner. In the Navrongo Health Research Center, we greatly appreciate the assistance of A. Bugri, S. Abudulai, and A. Wahab in the laboratory; all nurses and health workers in the War Memorial Hospital, Navrongo and the Health Centers of the KND; C. Tindana with all field workers and drivers for excellent work in the field; and T. Tei and M. Bugase for logistic support. We acknowledge the use of the Navrongo Demographic Surveillance System database, and we thank all study participants for their trust and contribution.”

